# MISIM v2.0: a web server for inferring microRNA functional similarity based on microRNA-disease associations

**DOI:** 10.1093/nar/gkz328

**Published:** 2019-05-09

**Authors:** Jianwei Li, Shan Zhang, Yanping Wan, Yingshu Zhao, Jiangcheng Shi, Yuan Zhou, Qinghua Cui

**Affiliations:** 1 Institute of Computational Medicine, School of Artificial Intelligence, Hebei University of Technology, Tianjin 300401, China; 2 Department of Biomedical Informatics, Department of Physiology and Pathophysiology, Center for Noncoding RNA Medicine, MOE Key Lab of Cardiovascular Sciences, School of Basic Medical Sciences, Peking University, 38 Xueyuan Rd, Beijing 100191, China; 3 Sanbo Brain Institute, Sanbo Brain Hospital, Capital Medical University, Beijing 100093, China

## Abstract

MicroRNAs (miRNAs) are one class of important small non-coding RNA molecules and play critical roles in health and disease. Therefore, it is important and necessary to evaluate the functional relationship of miRNAs and then predict novel miRNA-disease associations. For this purpose, here we developed the updated web server MISIM (**mi**RNA **sim**ilarity) v2.0. Besides a 3-fold increase in data content compared with MISIM v1.0, MISIM v2.0 improved the original MISIM algorithm by implementing both positive and negative miRNA-disease associations. That is, the MISIM v2.0 scores could be positive or negative, whereas MISIM v1.0 only produced positive scores. Moreover, MISIM v2.0 achieved an algorithm for novel miRNA-disease prediction based on MISIM v2.0 scores. Finally, MISIM v2.0 provided network visualization and functional enrichment analysis for functionally paired miRNAs. The MISIM v2.0 web server is freely accessible at http://www.lirmed.com/misim/.

## INTRODUCTION

MicroRNAs (miRNAs) are one class of important small non-coding RNA molecules which function as negative gene regulators by targeting mRNAs through base pairing (1,2). Given their important roles in various critical biological processes, it is well known that miRNAs are involved in a variety of human diseases, including cancer, cardiovascular diseases etc.(3), and thus could represent a novel therapeutic strategy (4–6). It was reported that miRNAs can be clustered as functional sets (7,8), suggesting that miRNAs could be functionally related with other miRNAs. Thus, it is important to quantify the functional relations among miRNAs and then build miRNA functional networks.

For this purpose, a number of computational methods have been developed (9–14), for example miRFunSim (11) evaluates miRNA functional similarity based on the topological properties of protein-protein interaction (PPI) network (15) associated with the paired miRNAs, the method proposed by Yu *et al.* and MiRGOFS evaluate miRNA functional similarity based on GO semantic similarity metric (16) associated with the paired miRNAs. The MISIM algorithm, developed by us in 2010, is based on the increasingly accumulated miRNA-disease association data (12). Functional similarity is defined as a relationship, for example co-expression similarity, co-GO similarity, co-literature similarity, and co-similar disease similarity. MISIM first calculates disease similarity based on MeSH disease terms and then quantifies miRNA functional similarity by integrating miRNA-disease association data and disease similarity scores. Now MISIM has become a frequently used platform for addressing issues of miRNA-disease association prediction (17) and drug development (18). A number of recent studies have addressed the prediction of miRNA-disease association based on the original MISIM algorithm. Researchers presented some computational models for miRNA-disease association prediction, such as the models of Laplacian Regularized Sparse Subspace Learning (19), Extreme Gradient Boosting Machine (20), Bipartite Network Projection (21), Matrix Decomposition and Heterogeneous Graph Inference (22). As eight years have passed, however, limitations emerged for the original MISIM method and other algorithms. Besides MISIM v1.0 was not updated using the latest miRNA-disease association data, one major limitation is that MISIM v1.0 only produces scores ≥0. All other algorithms also can only produce scores ≥0. However, it is well known that some miRNAs are negatively related in function. For example, some miRNA activate cell apoptosis, but some others inhibit cell apoptosis. Therefore, the functional similarity score could be negative but MISIM v1.0 and all other algorithms failed to do so. In addition, MISIM v1.0 only produces miRNA functional similarity scores but not provides options such as network visualization, functional analysis, and novel miRNA-disease association prediction. Further tasks should be performed to implement these options. For example, users can further use software of Pajek or Cytoscape to visualize the produced miRNA functional networks. For functional analysis, they may use software of TAM (7,23) or miEAA (24). An integration of these options will greatly facilitate users.

To overcome the above limitations, we developed the MISIM v2.0 web server. By improving the algorithm of MISIM v1.0, MISIM v2.0 can quantify miRNA functional similarity scores to positive, negative, and zero values. In addition, MISIM v2.0 runs on the latest miRNA-disease association dataset from HMDD v3.0 (3) to keep a pace with the rate of data accrual. Moreover, MISIM v2.0 implemented network visualization and functional analysis for selected miRNAs. Finally, MISIM v2.0 implemented an algorithm to predict novel miRNA-disease association. Different with previous miRNA-disease association prediction algorithm, MISIM v2.0 can predict directional miRNA-disease associations, that is, it can determine the association between a miRNA and a disease is positive or negative.

## METHODS

### Collection of datasets

We downloaded MeSH terms from the National Library of Medicine (http://www.nlm.nih.gov/). The human miRNA-disease association dataset was obtained from the HMDD v3.0 database (http://www.cuilab.cn/hmdd) (3). The disease names in HMDD were curated using MeSH disease terms. Finally, we obtained 14250 miRNA-disease associations, including 1044 miRNAs and 613 diseases. The miRNA-disease association data was curated using the latest version of miRBase (Release 22.1). We obtained 547 miRNA sets from TAM 2.0 (http://www.scse.hebut.edu.cn/tam/) (7). Furthermore, all the source code of MISIM v2.0 was freely available.

### Calculating miRNA functional similarity

**Figure 1.**
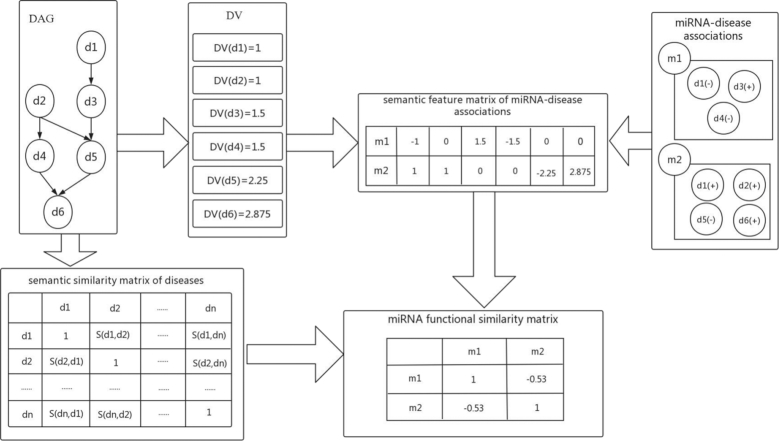
The flowchart of MISIM v2.0 algorithm.

The flowchart of MISIM v2.0 algorithm is shown in Figure 1. Firstly, we calculate the semantic value of a disease based on the MeSH disease structure of directed acyclic graph (DAG) with the method identical to the previous version of MISIM v1.0, ${\rm{DA}}{{\rm{G}}_d} = {\rm{\ }}( {{\rm{d}},{T_d},{E_d}}){\rm{\ }}$, Where d, ${T_d}$, ${E_d}$ represent disease d, the set of all ancestor nodes of d including d itself and the set of corresponding links, respectively. The semantic value ${D_d}( t )$ of disease d can be calculated based on the ${\rm{DA}}{{\rm{G}}_d}$.

(1) \begin{equation*}\left\{ {\begin{array}{@{}*{1}{l}@{}} {{D_d}\ \left( d \right) = \ 1}\nonumber \\ {{D_d}\ \left( t \right) = \max \left( {\Delta *{D_d}\left( {t^{\prime}} \right){\rm{|}}t^{\prime} \in children\ of\ t} \right)\ \ if\ t \ne d} \end{array}} \right.\\ \end{equation*}

where Δ is the semantic contribution factor for edges ${E_d} {\rm{\ }}$linking disease ${\rm{t}}$ with its child disease ${\rm{t^{\prime}}}$. Based on equation (1), we calculate the semantic value of disease d as follows:

(2) \begin{equation*}DV\ \left( d \right) = \mathop \sum \limits_{t \in {T_d}} {D_d}\left( t \right)\ \end{equation*}

The semantic similarity of two diseases is calculated in turn based on both the addresses of these diseases in DAG graphs and their semantic relations.

(3) \begin{equation*}{{DS\ \left({{d_1},{d_2}} \right)}} = \frac{{\mathop \sum \nolimits_{t\in {T_{{d_1}}}\mathop \cap {T_{{d_2}}}} \left( {{D_{{d_1}}}\left( t \right) + {D_{{d_2}}}\left( t \right)} \right)}}{{DV\left( {{d_1}} \right) + DV\left( {{d_2}} \right)}}\ \end{equation*}

After the above steps, the semantic similarity matrix of diseases is gained in the end and it is convenient to fetch the semantic similarity between arbitrary two diseases.

Secondly, it is known that miRNAs associated with similar diseases may have similar functions. Based on the miRNA-disease association from the datasets in MISIM v2.0, the matrix of semantic feature of the miRNA-disease association data is calculated. Moreover, we have in-depth curation of miRNA-disease associations by discriminating the up-regulated (positive) miRNAs and down-regulated (negative) miRNAs.

To better describe the method representing the up-regulated and down-regulated relations between miRNAs and diseases, we improve the formula (3), the semantic value of disease d is defined as

(4) \begin{equation*}r\_DV\ \left( {\rm{d}} \right) = {\left( { - 1} \right)^r}\ \mathop \sum \limits_{t \in {T_d}} {D_d}\left( t \right){\rm{\ \ }}\end{equation*}

where *r* represents the original deregulation types (up or down regulation), which has two possible values, +1 for down-regulated miRNAs and 0 for up-regulated miRNAs. Based on the above formulas, the miRNA-disease associations can be quantified as semantic feature vectors, which is defined as

(5) \begin{equation*}{f_{mi}} = {\rm{\ }}\left\{ {r\_DV\left( {{d_{{\rm{i}}1}}} \right),r\_DV\left( {{d_{{\rm{i}}2}}} \right), \ldots ,r\_DV\left( {{d_{in}}} \right)} \right\}\end{equation*}

where *n* is the total number of diseases associated with miRNA *mi*. Formula (5) reflects the global semantic feature of the diseases regulated by one miRNA in the MeSH disease structure of DAG. A bigger absolute value of one component in the disease vectors indicates the corresponding disease of this component includes more semantic information. It also means that the corresponding disease is more specific than other diseases which are regulated by the same miRNA.

At last, when the up-regulated and down-regulated miRNA-disease associations are considered, the difference of directions between two semantic feature vectors of miRNAs *m1* and *m2* should be taken into account when computing miRNA functional similarity. Then the association score vectors of the input miRNAs *m1* and *m2* can be calculated by using cosine correlation as:

(6) \begin{equation*}CO{S_{mi{\rm{r}}}}\ \left( {m1,m2} \right) = \ \frac{{{f_{m1}}f_{m2}^T}}{{\parallel {f_{m1}}\ \parallel \,\parallel {f_{m2}}\parallel \ }}\end{equation*}

When calculating miRNA functional similarity, we found a common phenomenon. The value of cosine correlation is often zero when the intersection of two disease sets of *m1* and *m2* is empty. However, we observed that there exist associations among MeSH disease structures of *m1* and *m2*. This means that the value of cosine correlation should not be zero in this case.

Based on the above observation, formula (3) is adopted to calculate the semantic similarity of any two diseases associated with *m1* and *m2*. Next, the disease sets associated with *m1* and *m2* are named as *D1* and *D2*. Then, the disease semantic similarity of *m1* and *m2* is calculated by formula (7).

(7) \begin{equation*}{f_{MDS\left( {m1,m2} \right)}} = \ \left\{ {DS\left( {{d_{11}},{d_{21}}} \right),DS\left( {{d_{12}},{d_{21}}} \right), \ldots ,DS\left( {{d_{1{\rm{n}}}},{d_{2{\rm{m}}}}} \right)} \right\}\end{equation*}

Then, the disease semantic features of *m*1 and *m*2 are improved by the following formulas respectively:

(8) \begin{equation*}{f_{m1}} = {\rm{\ }}\{ {f_{m1}},{f_{MDS\left( {m1,m2} \right)}}\} \end{equation*}

(9) \begin{equation*}{f_{m2}} = {\rm{\ }}\{ {f_{m2}},{f_{MDS\left( {m2,m1} \right)}}\} \end{equation*}

Finally, miRNA functional similarity will be calculated using formula (6) based on the novel disease semantic features calculated using formulas (8) and (9).

### Predicting novel miRNA-disease associations

Moreover, MISIM v2.0 can also be used to predict novel miRNA-disease associations and to infer novel potential functions or associated diseases for given miRNAs. It provides two predicting methods, one is ‘miRNA-Disease Association’, and the other one is ‘miRNA-Disease Association for all’.

The method of ‘miRNA-Disease Association’ is to predict the novel miRNA-disease associations of two miRNAs, *m*1 and *m*2. The associated diseases sets of *m*1 and *m*2 are named *D*1 and *D*2, respectively. Then the intersection of *D*1 and *D*2 is calculated and is named as *D*3. Next, MISIM v2.0 calculates the subtraction between *D*1 and *D*3 and then the the subtraction will be considered to be the predicted novel disease associated with *m*2. Moreover, the reciprocals of the disease semantic values are treated as the probability of potential novel diseases. A higher probability means the prediction result is more reliable. Normally, the general disease has a higher reciprocal of the semantic value.

Correspondingly, the method of ‘miRNA-Disease Association for all’ is to predict the novel miRNA-disease associations in the total datasets for single miRNA submitted by the user. In the process of prediction, the miRNA *m*1 submitted by the user is calculated with the miRNA *mi* ($mi \ne m1$) in the dataset one by one. The associated disease sets of *m*1 and *mi* are named *D*1 and *Di*, the intersection of *D*1 and *Di* was *D3*. The subtraction of *Di* and *D*3 will be the predicted novel miRNA-disease associations of *m*1. MISIM v2.0 takes the values of miRNA functional similarity as weighting factors of this subtraction, and calculates the weighted frequencies of each novel disease.

### miRNA functional enrichment analysis

For calculated functionally paired miRNAs or miRNAs in a constructed miRNA functional network, it is interesting to dissect the enriched functions of these miRNAs. For doing so, we implemented the algorithm of TAM 2.0 (7) in MISIM v2.0.

### Visualization of miRNA functional networks

We implemented the visualization of miRNA functional networks by visjs + G2. Users can change node shape (e.g. box, diamond, triangle, ellipse), node size, and set the threshold of functional similarity value for whether there is a link between two miRNAs. In addition, if the users choose an option for directional functional similarity, the positive links (functional similarity with positive value) and negative links (functional similarity with negative value) will be in different colors. Once the network was finalized, users also can download the network picture.

### Server construction

We built the MISIM v2.0 web server in the ‘Linux + Apache + MySQL + PHP’ framework. The data visualization was implemented by using open source visjs package (http://visjs.org/network_examples.html) and G2 package (http://npm.taobao.org/package/g2).

## RESULTS

### Overview of MISIM v2.0 web server

Currently, MISIM v2.0 contains 1044 miRNAs and 613 diseases, a 3-fold increases compared to MISIM v1.0. More importantly, it collects information that how deregulation (up-regulated or down-regulated) of a miRNA is involved in a corresponding disease.

MISIM v2.0 works as the following flowchart. Firstly, for analysis, users have four options, ‘ALL vs ALL Similarity’ (which supports to calculate the paired functional similarity values for a group of miRNAs), ‘One vs ALL Similarity’ (which supports to calculate the paired functional similarity values for a single miRNA with all other miRNAs), ‘miRNA-Disease Association’ (which predicts the associated disease for a paired miRNAs by evaluating the functional relations of the two miRNAs), and ‘Prediction miRNA disease association for all’ (which predicts the associated disease for a single miRNA by evaluating the functional relations of the given miRNA with all other miRNAs). In addition, the calculated miRNA functional similarity values can be downloaded in the ‘Download’ menu. For the option of ‘ALL vs ALL Similarity’, the users need to first input candidate miRNA list (one miRNA in each line). Then they can check or uncheck ‘Considering up/down-regulation in the similarity calculation’ box. If they leave this box unchecked, MISIM v2.0 will not consider the direction of miRNA deregulation. A ‘Considering up/down-regulation in the similarity calculation’ option means MISIM v2.0 will consider up- or down- regulation of miRNAs in associated diseases. Finally, when ‘Submit’ was clicked, the calculated functional similarity scores of paired miRNAs of the inputted miRNAs will be shown. For the calculated results, the users can further perform network visualization and functional enrichment analysis. Moreover, MISIM v2.0 can be also used to predict the potential novel miRNA-Disease associations of input miRNAs.

### Example for analyzing functional similarity for a list of miRNAs

**Figure 2.**
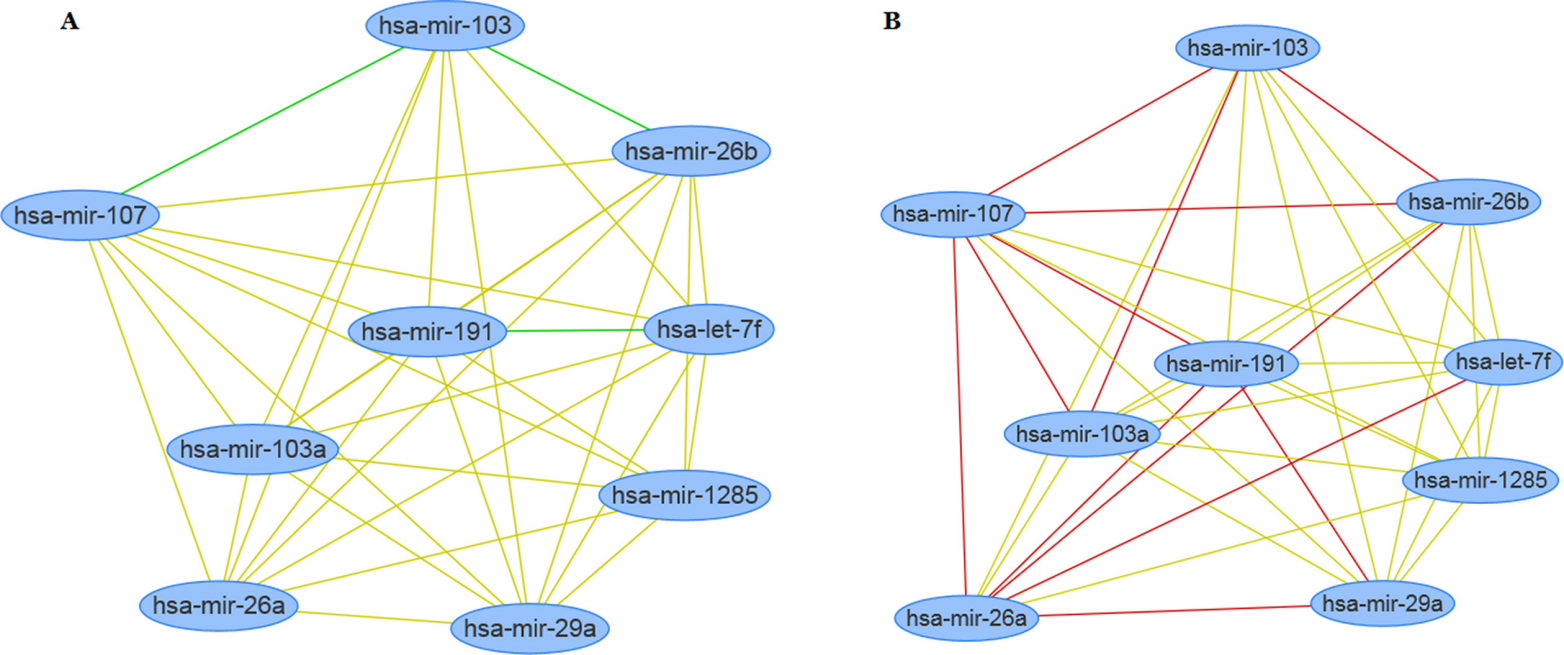
The visualization of the constructed miRNA functional networks using MISIM v2.0 web server and example miRNA list. (**A**) miRNA functional network with negative links produced using the option ‘Considering up/down-regulation in the similarity calculation’. (**B**) miRNA functional network without negative links produced using the option ‘default’.

To show this function of MISIM v2.0 web server, we click the menu of ‘ALL versus ALL Similarity’. Then we used the sample miRNA list. Next we select the ‘Considering up/down-regulation in the similarity calculation’ option in step 2. After clicking ‘Submit’ button, the result was thus shown in the right panel of the page. We noted that there are positive and negative functional similarity values for paired miRNAs. For example, the functional similarity value between hsa-let-7f and hsa-mir-107 is 0.48628593, whereas that between hsa-mir-107 and hsa-mir-103 is −0.19892149. The users can further download the calculated results, visualize these miRNAs and functional similarity values as a network, and perform functional enrichment analysis. The calculated miRNA functional network is shown as Figure 2A. If the users select the default option, that is, no consideration for the up-/down-regulation of miRNAs in disease, the network will be as Figure 2B.

### Example for predicting associated disease for a miRNA

To show this function of MISIM v2.0 web server, we click the menu of ‘Prediction miRNA disease association for all’. We use hsa-mir-29 as an example and select ‘Considering up/down-regulation in the similarity calculation’ in the second step. After clicking ‘Predict’, the predicted associated diseases were shown in the down panel of this page. As a result, 570 novel diseases associated with hsa-mir-29 were predicted. For each predicted disease, the significance and possible deregulation direction were also given. For example, MISIM v2.0 predicted that hsa-mir-29 is negatively associated with Mental Disorders (Weighted frequency = −0.26). Although we did not find any experimental confirmations for this prediction in PubMed but found a literature, which reported that hsa-mir-29 was significantly decreased in the serum of patients with Alzheimer's disease. Given that Mental Disorders and Alzheimer's disease are highly related (25), it could be believable that hsa-mir-29 is also generally negatively involved in Mental Disorders. In addition, we can download all the predicted results.

## CONCLUSION

In conclusion, we developed MISIM v2.0, which significantly improved the original MISIM algorithm. For a list miRNAs, MISIM v2.0 will calculate the functional similarity for all paired miRNAs. For one single miRNA, MISIM v2.0 will calculate the functional similarity of the given miRNAs with all other miRNAs one by one. For both the above options, MISIM v2.0 can produce the miRNA functional networks and perform miRNA functional enrichment analysis for the investigated miRNAs. In addition, MISIM v2.0 also can predict novel miRNA-disease associations which could be non-directional or directional.

Of course, although MISIM v2.0 was improved significantly compared with MISIM v1.0, limitations still exist. One limitation is that the miRNA-disease association data is incomplete, which could produce bias when calculating miRNA functional similarity. Given that a number of computational methods for the prediction of novel miRNA-disease association are based on MISIM scores. Thus, there should be bias for the prediction of novel miRNA-disease association. Therefore, integrating other methods, for example those based on GO terms or PPI network topologies, could improve the calculation of miRNA functional similarity. Another limitation is that the network visualization of MISIM v2.0 web server could do not work well for some internet browser. The third limitation is that currently MISIM v2.0 only runs for pre-miRNAs and cannot discriminate between specific 5p and 3p mature miRNAs. The reason is that HMDD annotated miRNA-disease data at the level of miRNA precursor. We could improve HMDD and thus MISIM v2.0 by considering mature miRNAs as well. The fourth limitation is that currently MISIM v2.0 only runs for human miRNAs but cannot be applied to miRNAs from other species. The reason is also the HMDD database only contain human data. We will collect miRNA–disease association data from other species, for example, mouse and rat, and then update MISIM as well in the future. We will continue testing MISIM v2.0 in different internet browsers to make it more convenient to users.

## References

[1] Ambros V. The functions of animal microRNAs. Nature. 2004; 431:350–355. 15372042 10.1038/nature02871

[2] Bartel D.P. MicroRNAs: genomics, biogenesis, mechanism, and function. Cell. 2004; 116:281–297. 14744438 10.1016/s0092-8674(04)00045-5

[3] Huang Z., Shi J., Gao Y., Cui C., Zhang S., Li J., Zhou Y., Cui Q. HMDD v3.0: a database for experimentally supported human microRNA–disease associations. Nucleic Acids Res. 2019; 47:D1013–D1017. 30364956 10.1093/nar/gky1010 PMC6323994

[4] Matsui M., Corey D.R. Non-coding RNAs as drug targets. Nat. Rev. Drug. Discov. 2017; 16:167–179. 27444227 10.1038/nrd.2016.117 PMC5831170

[5] Rupaimoole R., Slack F.J. MicroRNA therapeutics: towards a new era for the management of cancer and other diseases. Nat. Rev. Drug. Discov. 2017; 16:203–222. 28209991 10.1038/nrd.2016.246

[6] Warner K.D., Hajdin C.E., Weeks K.M. Principles for targeting RNA with drug-like small molecules. Nat. Rev. Drug. Discov. 2018; 17:547–558. 29977051 10.1038/nrd.2018.93 PMC6420209

[7] Li J., Han X., Wan Y., Zhang S., Zhao Y., Fan R., Cui Q., Zhou Y. TAM 2.0: tool for microRNA set analysis. Nucleic Acids Res. 2018; 46:W180–W185. 29878154 10.1093/nar/gky509 PMC6031048

[8] Lu M., Zhang Q., Deng M., Miao J., Guo Y., Gao W., Cui Q. An analysis of human microRNA and disease associations. PLoS One. 2008; 3:e3420. 18923704 10.1371/journal.pone.0003420 PMC2559869

[9] Ding P., Luo J., Xiao Q., Chen X. A path-based measurement for human miRNA functional similarities using miRNA–disease associations. Sci. Rep. 2016; 6:32533. 27585796 10.1038/srep32533 PMC5009308

[10] Liu Y., Zeng X., He Z., Zou Q. Inferring microRNA–disease associations by random walk on a heterogeneous network with multiple data sources. IEEE/ACM Trans. Comput. Biol. Bioinf. 2017; 14:905–915. 10.1109/TCBB.2016.2550432 27076459

[11] Sun J., Zhou M., Yang H., Deng J., Wang L., Wang Q. Inferring potential microRNA-microRNA associations based on targeting propensity and connectivity in the context of protein interaction network. PLoS One. 2013; 8:e69719. 23874989 10.1371/journal.pone.0069719 PMC3713046

[12] Wang D., Wang J., Lu M., Song F., Cui Q. Inferring the human microRNA functional similarity and functional network based on microRNA-associated diseases. Bioinformatics. 2010; 26:1644–1650. 20439255 10.1093/bioinformatics/btq241

[13] Yang Y., Fu X., Qu W., Xiao Y., Shen H.B. MiRGOFS: a GO-based functional similarity measurement for miRNAs, with applications to the prediction of miRNA subcellular localization and miRNA–disease association. Bioinformatics. 2018; 34:3547–3556. 29718114 10.1093/bioinformatics/bty343

[14] Yu G., Xiao C.L., Bo X., Lu C.H., Qin Y., Zhan S., He Q.Y. A new method for measuring functional similarity of microRNAs. J. Integrated OMICS. 2011; 1:49–54.

[15] Wang Q., Sun J., Zhou M., Yang H., Li Y., Li X., Lv S., Li X., Li Y. A novel network-based method for measuring the functional relationship between gene sets. Bioinformatics. 2011; 27:1521–1528. 21450716 10.1093/bioinformatics/btr154

[16] Xu Y., Guo M., Shi W., Liu X., Wang C. A novel insight into Gene Ontology semantic similarity. Genomics. 2013; 101:368–375. 23628645 10.1016/j.ygeno.2013.04.010

[17] Chen X., Wang L., Qu J., Guan N.N., Li J.Q. Predicting miRNA–disease association based on inductive matrix completion. Bioinformatics. 2018; 34:4256–4265. 29939227 10.1093/bioinformatics/bty503

[18] Liang X., Zhang P., Yan L., Fu Y., Peng F., Qu L., Shao M., Chen Y., Chen Z. LRSSL: predict and interpret drug-disease associations based on data integration using sparse subspace learning. Bioinformatics. 2017; 33:1187–1196. 28096083 10.1093/bioinformatics/btw770

[19] Chen X., Huang L. LRSSLMDA: Laplacian Regularized Sparse Subspace Learning for MiRNA-Disease Association prediction. PLoS Comput. Biol. 2017; 13:e1005912. 29253885 10.1371/journal.pcbi.1005912 PMC5749861

[20] Chen X., Huang L., Xie D., Zhao Q. EGBMMDA: Extreme Gradient Boosting Machine for MiRNA-Disease Association prediction. Cell Death Dis. 2018; 9:3. 29305594 10.1038/s41419-017-0003-x PMC5849212

[21] Chen X., Xie D., Wang L., Zhao Q., You ZH., Liu H. BNPMDA: Bipartite Network Projection for MiRNA-Disease Association prediction. Bioinformatics. 2018; 34:3178–3186. 29701758 10.1093/bioinformatics/bty333

[22] Chen X., Yin J., Qu J., Huang L. MDHGI: Matrix Decomposition and Heterogeneous Graph Inference for miRNA–disease association prediction. PLoS Comput. Biol. 2018; 14:e1006418. 30142158 10.1371/journal.pcbi.1006418 PMC6126877

[23] Lu M., Shi B., Wang J., Cao Q., Cui Q. TAM: a method for enrichment and depletion analysis of a microRNA category in a list of microRNAs. BMC Bioinformatics. 2010; 11:419. 20696049 10.1186/1471-2105-11-419 PMC2924873

[24] Backes C., Khaleeq Q.T., Meese E., Keller A. miEAA: microRNA enrichment analysis and annotation. Nucleic Acids Res. 2016; 44:W110–W116. 27131362 10.1093/nar/gkw345 PMC4987907

[25] Ma W., Wu M., Zhou S., Tao Y., Xie Z., Zhong Y. Reduced Smoothened level rescues Abeta-induced memory deficits and neuronal inflammation in animal models of Alzheimer's disease. J. Genet. Genomics. 2018; 45:237–246. 29807798 10.1016/j.jgg.2018.05.001

